# Sex Chromosome Evolution and Genomic Divergence in the Fish *Hoplias malabaricus* (Characiformes, Erythrinidae)

**DOI:** 10.3389/fgene.2018.00071

**Published:** 2018-03-05

**Authors:** Alexandr Sember, Luiz A. C. Bertollo, Petr Ráb, Cassia F. Yano, Terumi Hatanaka, Ezequiel A. de Oliveira, Marcelo de Bello Cioffi

**Affiliations:** ^1^Laboratory of Fish Genetics, Institute of Animal Physiology and Genetics, Czech Academy of Sciences, Liběchov, Czechia; ^2^Departamento de Genética e Evolução, Universidade Federal de São Carlos, São Carlos, Brazil; ^3^Secretaria de Estado de Educação de Mato Grosso (SEDUC-MT), Cuiabá, Brazil

**Keywords:** fish cytogenetics, multiple sex chromosomes, sex-determining region, sex chromosome turnover, CGH, intraspecific variability, species complex, speciation

## Abstract

The Erythrinidae family (Teleostei: Characiformes) is a small Neotropical fish group with a wide distribution throughout South America, where *Hoplias malabaricus* corresponds to the most widespread and cytogenetically studied taxon. This species possesses significant genetic variation, as well as huge karyotype diversity among populations, as reflected by its seven major karyotype forms (i.e., karyomorphs A-G) identified up to now. Although morphological differences in their bodies are not outstanding, *H*. *malabaricus* karyomorphs are easily identified by differences in 2*n*, morphology and size of chromosomes, as well as by distinct evolutionary steps of sex chromosomes development. Here, we performed comparative genomic hybridization (CGH) to analyse both the intra- and inter-genomic status in terms of repetitive DNA divergence among all but one (E) *H. malabaricus* karyomorphs. Our results indicated that they have close relationships, but with evolutionary divergences among their genomes, yielding a range of non-overlapping karyomorph-specific signals. Besides, male-specific regions were uncovered on the sex chromosomes, confirming their differential evolutionary trajectories. In conclusion, the hypothesis that *H*. *malabaricus* karyomorphs are result of speciation events was strengthened.

## Introduction

The Erythrinidae family (Teleostei: Characiformes) is a small group of Neotropical fishes with a wide distribution throughout South America. This family currently consists of three well-recognized genera—*Erythrinus* (Scopoli, 1777), *Hoplias* (Gill, 1903), and *Hoplerythrinus* (Gill, 1895) with at least 15 until now recognized species (Oyakawa, 2003; Oyakawa and Mattox, 2009). Erythrinids live in diverse habitats, from small lakes and lagoons to large rivers (Oyakawa, 2003). However, unlike the large migratory Neotropical fishes, they are usually not able to overcome obstacles such as waterfalls and large rapids, due to their sedentary lifestyle (Oyakawa, 2003). This situation may have contributed to reduced gene flow between sub-populations in the same hydrographic basin. Consequently, great genome diversity has been documented within the Erythrinidae family, reflected in the noticeable diversity of karyotypes—particularly in the diploid number (2*n*), karyotype structure and sex chromosome systems (reviewed in Bertollo, 2007).

*Hoplias malabaricus* is the most widespread as well as cytogenetically investigated taxon, with analyzed populations from north to south of Brazil, Uruguay, Argentina, and Suriname. Despite its wide distribution, this taxon is characterized by low vagility, tending to constitute small populations, being able to survive under low oxygen conditions and to adapt to new environments (Rantin et al., 1992, 1993; Rios et al., 2002). Such characteristics are probably associated with the significant genetic variation and enormous karyotype diversity evidenced by the seven major karyotype forms (i.e., karyomorphs A-G Bertollo et al., 2000; Cioffi et al., 2012). However, despite rather morphological uniformity of body plan, *H*. *malabaricus* karyomorphs are easily distinguished by 2*n*, morphology and size of chromosomes, as well as by different evolutionary stages of distinct sex chromosome systems, indicating the occurrence of an unrecognized species diversity (Bertollo et al., 2000; Bertollo, 2007). Among the seven karyomorphs examined to date, only those of A and E do not show heteromorphic sex chromosomes. Indeed, a well-differentiated XY sex chromosome system occurs in the karyomorph B, while karyomorphs C and F possess such system in an early state of differentiation and finally, karyomorphs D and G harbor a X_1_X_2_Y and XY_1_Y_2_ multiple sex system, respectively (reviewed in Freitas et al., 2017). These findings indicate independent origins of sex chromosome systems as evidenced by whole chromosome painting (WCP) data (Cioffi et al., 2013). Altogether, *H. malabaricus* provides a suited model for evolutionary, cytotaxonomic and biodiversity analyses (for review see Cioffi et al., 2012).

The development of recent molecular methologies has allowed a qualitative improvement on chromosome researches of different biological taxa. Among them, the genomic *in situ* hybridization (GISH) and comparative genomic hybridization (CGH), originally developed for clinical studies (Kallioniemi et al., 1992), are now successfully applied for several other purposes, such as the identification of parental genomes in hybrids/allopolyploids (Bi and Bogart, 2006; Knytl et al., 2013; Symonová et al., 2013a, 2015; Doležálková et al., 2016; Majtánová et al., 2016), the detection of sex-specific content on homomorphic sex chromosomes (Ezaz et al., 2006; Altmanová et al., 2016; Rovatsos et al., 2016; Freitas et al., 2017) and the genome comparisons among related species (Valente et al., 2009; Symonová et al., 2013b; Majka et al., 2016; Carvalho et al., 2017; Moraes et al., 2017). All these (and many other) studies proved that GISH and CGH technologies, despite representing rather “rough” molecular tools, may be successful in providing clues about the genome evolution, with their resolution being based on the differential distribution of already divergent genome-specific repetitive DNA classes (Kato et al., 2005; Chester et al., 2010), as these sequences are generally highly abundant in eukaryotic genomes and display faster evolutionary rates than the single-copy ones (Charlesworth et al., 1994; Cioffi and Bertollo, 2012; López-Flores and Garrido-Ramos, 2012). Hence, in the present study, we performed analyses including CGH procedures to explore both inter- and intra-genomic divergences (within the range defined above) among *H. malabaricus* karyomorphs. Our results provided new insigths for better understanding of the ongoing processes of the karyomorph differentiations, as well as their sex chromosome systems.

## Materials and methods

### Individuals and mitotic chromosome preparations

Analyzed representatives of *H. malabaricus* karyomorphs are given in Table 1. The individuals were collected under appropriate authorization of the Brazilian environmental agency ICMBIO/SISBIO (License numbers 48628-2 and 10538-1) and deposited in the fish collection of the Cytogenetic Laboratory, Departamento de Genética e Evolução, Universidade Federal de São Carlos. Mitotic chromosomes were obtained by protocols described in Bertollo et al. (2015). The experiments followed ethical and anesthesia conducts, in accordance with the Ethics Committee on Animal Experimentation of the Universidade Federal de São Carlos (Process number CEUA 1853260315).

**Table 1:** Collection sites of *Hoplias malabaricus* karyomorphs, with the respective localities and sample sizes.

***Hoplias malabaricus* karyomorphs**	**Locality**	***N***	**References**
Karyomorph A	Pântano River (SP)	08♂ 06♀	Cioffi et al., 2009
Karyomorph B	Doce River (MG)	07♂ 08♀	Cioffi et al., 2010
Karyomorph C	Bento Gomes River (MT)	11♂ 09♀	Cioffi and Bertollo, 2010
Karyomorph D	UFSCar reservoir: Monjolinho Stream (SP)	10♂ 07♀	Cioffi and Bertollo, 2010
Karyomorph F	São Francisco River (MG)	12♂ 11♀	Freitas et al., 2017
Karyomorph G	Aripuanã (MT)—Aripuanã River	12♂ 11♀	Oliveira et al., 2018

*AM, Amazonas; SP, São Paulo; MT, Mato Grosso; MG, Minas Gerais States*.

### Preparation of probes for comparative genomic hybridization (CGH)

**Figure 1:**
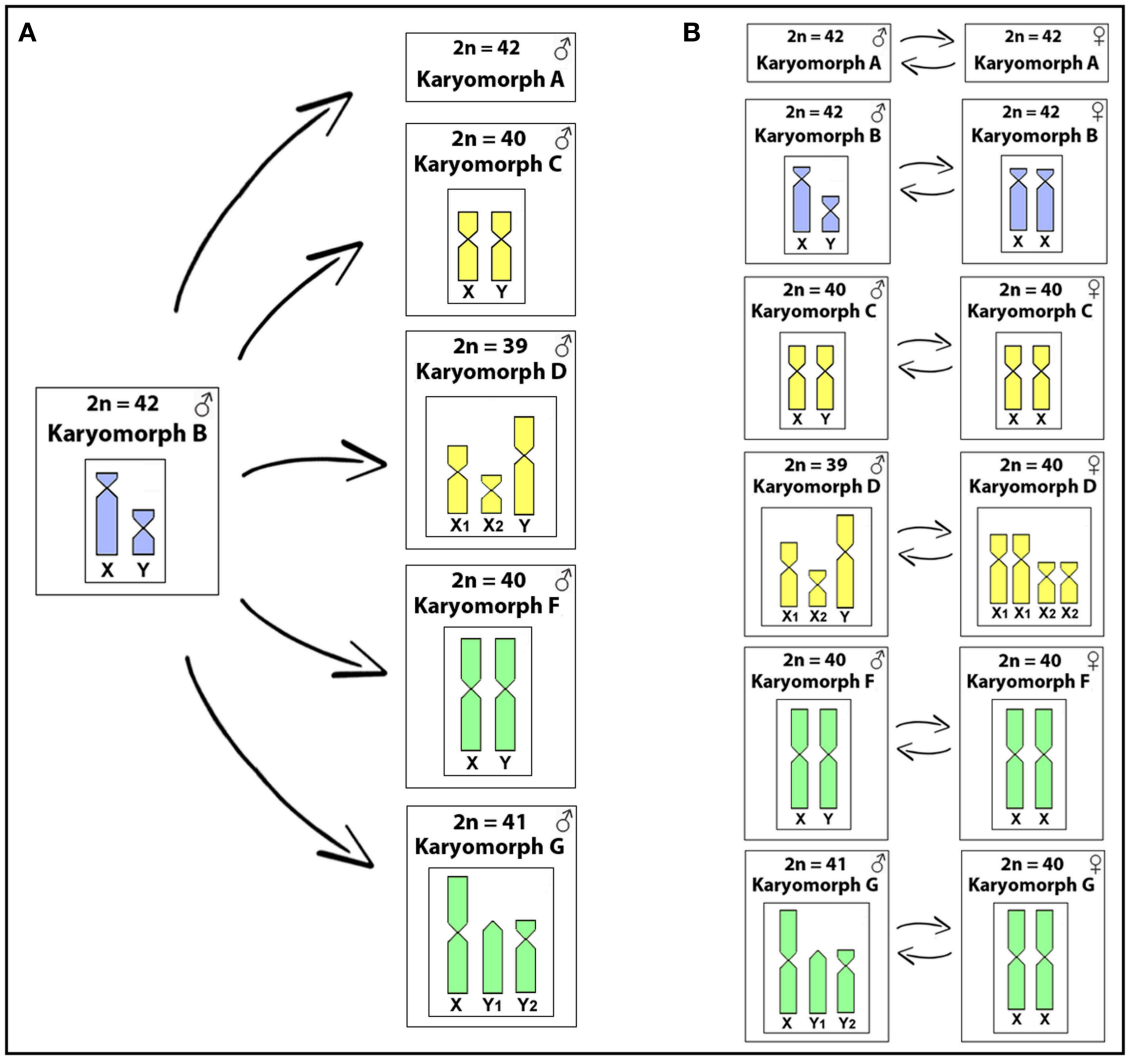
The experimental designs used in this study. In the first one **(A)**, gDNA of karyomorph B male specimens was used for hybridization against chromosomal background of the other karyomorphs (except for karyomorph E), focusing on inter-karyomorph comparisons. In the second set of experiments **(B)** male- and female-derived gDNAs of each karyomorph under study were hybridized together, focusing on the intra-karyomorph comparisons, with special emphasis on the molecular composition of putative, nascent, or well-differentiated sex chromosomes.

The total genomic DNAs (gDNAs) from male and female specimens of all karyomorphs listed in Table 1 were extracted from liver tissue by the standard phenol-chloroform-isoamylalkohol method (Sambrook and Russell, 2001). Two different experimental designs were used for this study, as outlined in Figure 1. In the first set of experiments (inter-karyomorph genomic comparisons), the gDNA of karyomorph B male specimens was chosen as a reference due to its well-differentiated XY sex chromosomes and used for hybridization against metaphase chromosomes of the other karyomorphs (Figure 1A). For this purpose, male-derived gDNAs of each karyomorph A, C, D, F and G were labeled with digoxigenin-11-dUTP using DIG-nick-translation Mix (Roche, Mannheim, Germany), while male-derived gDNA of karyomorph B was labeled with biotin-16-dUTP using BIO-nick-translation Mix (Roche). For blocking the repetitive sequences in all experiments, we used unlabeled C_0_t-1 DNA (i.e., fraction of genomic DNA enriched for highly and moderately repetitive sequences), prepared according to Zwick et al. (1997). Hence, the final probe cocktail for each slide was composed of 500 ng of male-derived gDNA of karyomorph B, 500 ng of male-derived DNA corresponding to one of the comparative karyomorphs, 15 μg of female-derived C_0_t-1 DNA of karyomorph B and 15 μg of female-derived C_0_t-1 DNA from the respective comparative karyomorph. The probe was ethanol-precipitated and the dry pellets were resuspended in hybridization buffer containing 50% formamide, 2 × SSC, 10% SDS, 10% dextran sulfate and Denhardt's buffer, pH 7.0. In the second set of experiments (Figure 1B) we focused on intra-karyomorph comparisons, with special emphasis on molecular composition of putative, nascent or well-differentiated sex chromosomes. In this case, male-derived gDNAs of all karyomorphs were labeled with biotin-16-dUTP and female gDNAs with digoxigenin-11-dUTP by means of nick translation as described above. The final hybridization mixture for each slide (20 μl) was composed of male- and female-derived gDNAs (500 ng each), 25 μg of female-derived C_0_t-1 DNA from the respective karyomorph and the hybridization buffer described above. The chosen ratio of probe vs. C_0_t-1 DNA amount was based on the experiments performed in our previous studies in fishes including erythrinids (Symonová et al., 2013a,b, 2015; Carvalho et al., 2017; Freitas et al., 2017; Moraes et al., 2017; Yano et al., 2017; Oliveira et al., 2018) and corroborated the ratio used in other related fish studies (e.g., Valente et al., 2009). According to our experiences, this ratio reflects high stringency towards repetitive DNA blocking and yet avoids the probability of improper probe dissolution in the hybridization buffer, which would otherwise cause artifacts.

### Fish used for CGH

The CGH experiments followed the methodology described in Symonová et al. (2015), with modifications. Briefly, prior to hybridization, slides were aged at 37°C for 2h, followed by an RNAse A (90 min, 37°C) and then pepsin (50 μg/ml in 10 mM HCl, 3 min, 37°C) treatments. Chromosomes were subsequently denatured in 75% formamide (pH 7.0) in 2 × SSC (74°C, 3 min), and then immediately cooled and dehydrated through 70% (cold), 85%, and 100% (RT) ethanol series. The hybridization mixture was denatured at 86°C for 6 min, cooled at 4°C (10 min) and then applied on the slides. The hybridization was performed at 37°C for 72 h. Post-hybridization washes were carried out once in 50% formamide in 2 × SSC (pH 7.0) (44°C, 10 min each) and three times in 1 × SSC (44°C, 7 min each). Prior to probe detection, the slides were incubated with 3% non-fat dried milk (NFDM) in order to avoid the non-specific binding of antibodies. The hybridization signal was detected using Anti-Digoxigenin-Rhodamin (Roche) and Avidin-FITC (Sigma). Chromosomes were counterstained and mounted in antifade containing 1.5 μg/ml DAPI (Vector, Burlingame, CA, USA).

### Microscopic analyses and image processing

At least 30 metaphase spreads per individual were analyzed to confirm the 2*n*, karyotype structure and CGH results. Images were captured using an Olympus BX50 microscope (Olympus Corporation, Ishikawa, Japan), with CoolSNAP and the images were processed using Image Pro Plus 4.1 software (Media Cybernetics, Silver Spring, MD, USA). Chromosome morphology was classified according to Levan et al. (1964).

## Results

### Inter-karyomorph genomic relationships

**Figure 2:**
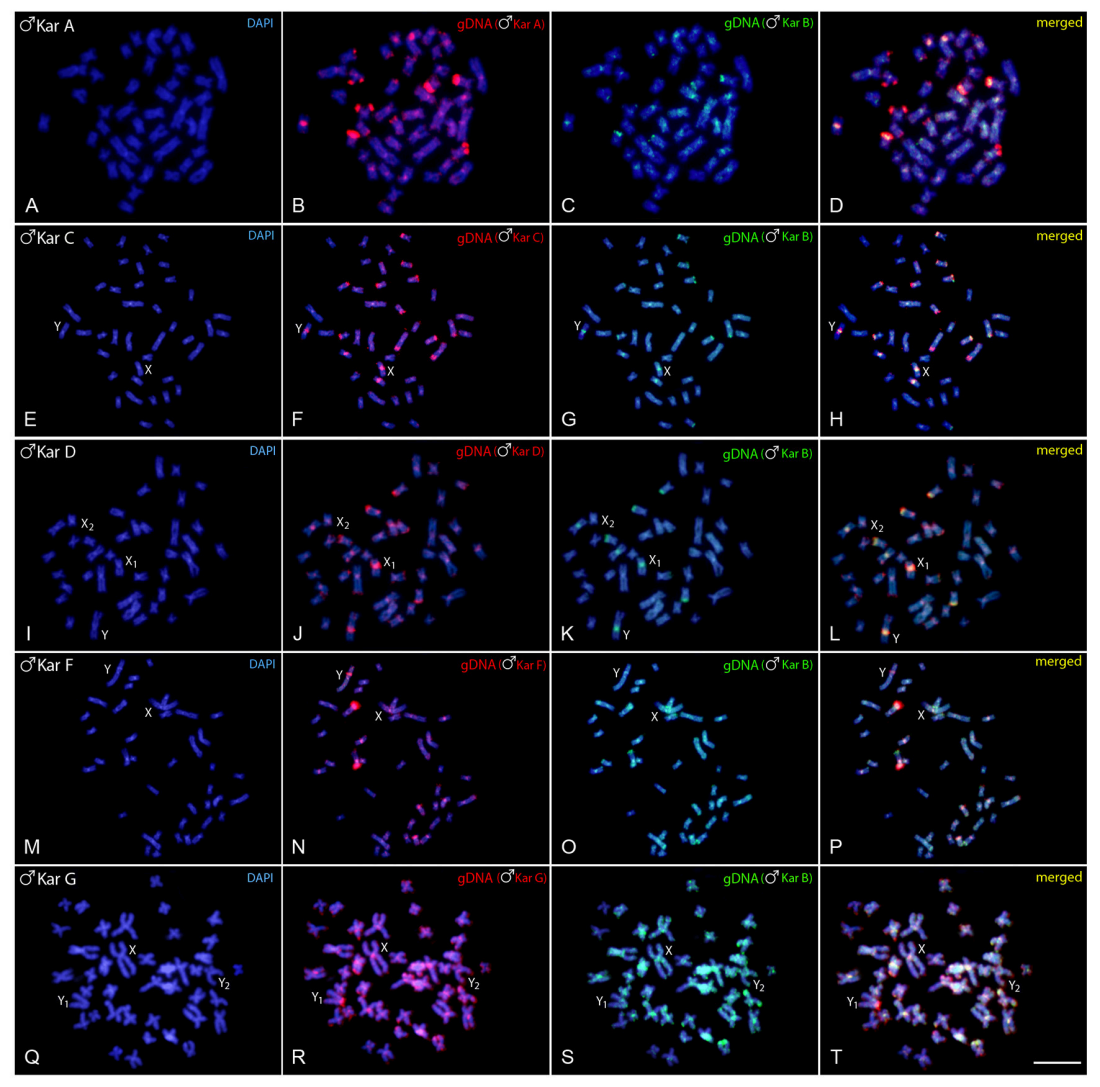
Mitotic chromosome spreads of *Hoplias malabaricus* males after CGH—interkaryomorph comparison. Male-derived genomic probe from karyomorph B mapped against male chromosomes of karyomorph A **(A–D)**, karyomorph C **(E–H)**, karyomorph D **(I–L)**, karyomorph F **(M–P)**, and karyomorph G **(Q–T)**. First column **(A,E,I,M,Q,I)**: DAPI images (blue); Second column **(B,F,J,N,R)**: hybridization pattern using the male-derived probe (red) of each analyzed karyomorph; Third column **(C,G,K,O,S)**: hybridization pattern using the male-derived probe of karyomorph B (green). Fourth column **(D,H,L,P,T)**: merged images of both genomic probes and DAPI staining. The common genomic regions of both compared karyomorphs are depicted in yellow. Bar = 10 μm.

In each experiment, both genome-derived probes showed rather equal binding to all chromosomes, with preferential localization in the centromeric and pericentromeric regions of most chromosomes and in terminal parts of some of them (yellow signals, i.e., combination of green and red), indicating the shared repetitive DNA content in such regions. The hybridization pattern in karyomorph A displayed stronger binding of the A-derived probe to the centomeric or telomeric regions of several chromosomes, while the B-derived probe that co-hybridized to these regions, produced signals of less intensity. Moreover, several exclusive A-specific markings appeared mostly in distal chromosomal regions (Figures 2A–D). Similar situation was observed also in karyomorph C (Figures 2E–H), where the majority of the accumulated blocks was shared by both probes, including those in the pericentromeric regions of the XY chromosomes, but several signals and especially those located in the terminal region of the long arms of the largest m pair were found to be accumulated with C-specific probe only. In karyomorph D, stronger binding of the D-derived probe was highlighted in many centromeres, in addition to some telomeric segments (Figures 2I–L). Remarkably, both genomic probes equally stained the heterochromatic block displayed by the neo-Y chromosome. In the karyomorph F, besides the shared binding pattern to majority of heterochromatic blocks, the F-derived probe yielded specific signals on the chromosomal pair bearing NOR-like regions (Figures 2M–P). In addition, the male-specific region on the nascent Y chromosome of this karyomorph displayed some affinity to B-derived male probe, despite being preferentially labeled with the F-male specific one (Figures 2N,O). In comparison to that, the male-specific region of karyomorph G, covering the entire short arms of the Y_1_ chromosome, was stained almost exclusively by the G-derived probe, while the B-derived probe produced only faint and dispersed signals in this region (Figures 2Q–T). In a similar way, the G-specific probe showed predominant binding to terminally located repetitive blocks on a few other chromosomes.

### Intra-karyomorph genomic relationships: detecting male-specific regions

Experiments performed on female chromosome spreads of karyomorphs B, C, D, F, and G showed the absence of identifiable sex-specific segments.

**Figure 3:**
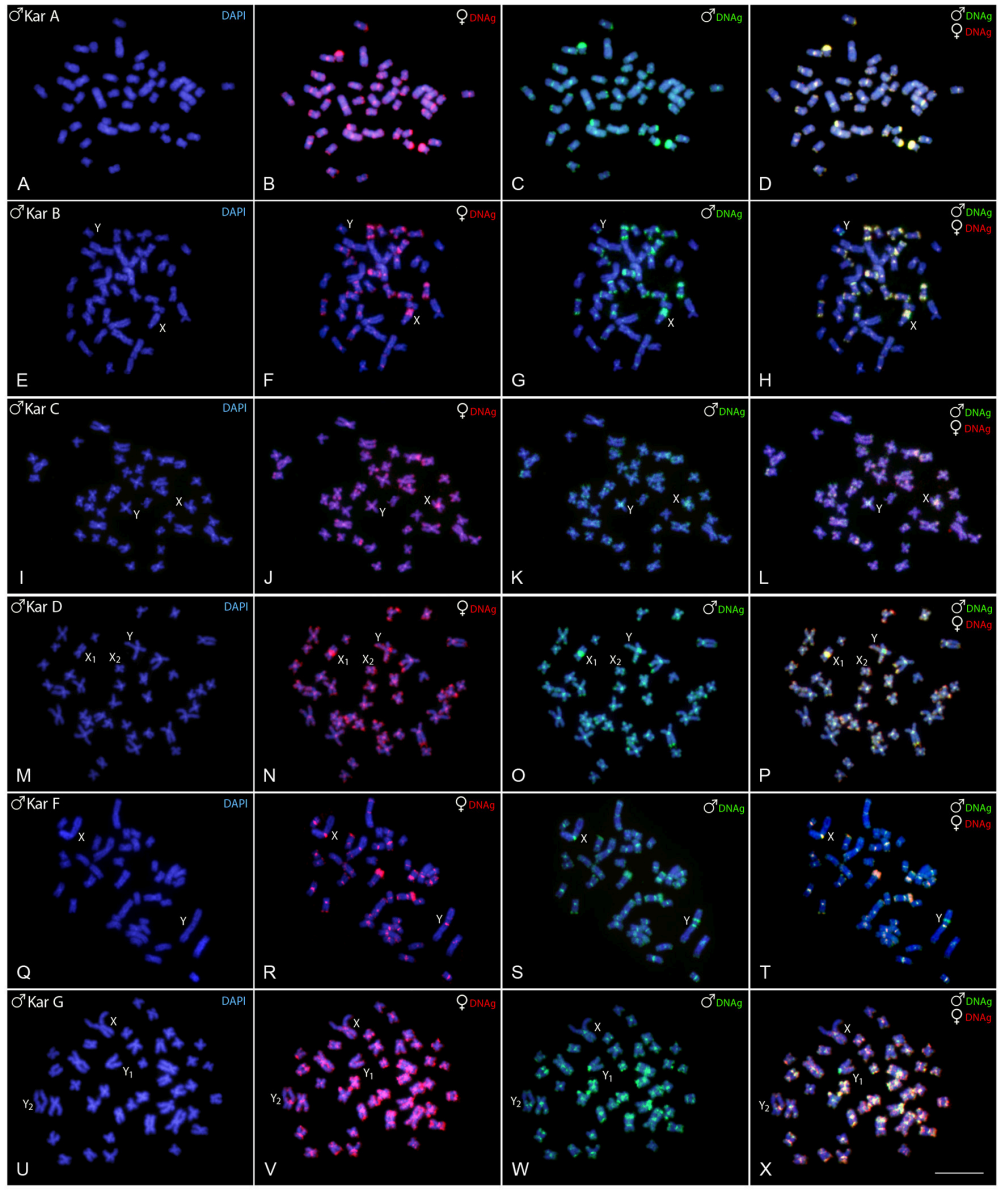
Mitotic chromosome spreads of *Hoplias malabaricus* males after CGH—intra-karyomorph hybridizations. Male- and female-derived genomic probes hybridized together for each karyomorph. First column **(A,E,I,M,Q,U)**: DAPI images (blue); Second column **(B,F,J,N,R,V)**: hybridization pattern of the female-derived probe (red) of each analyzed karyomorph; Third column **(C,G,K,O,S,W)**: hybridization pattern of the male-derived probe (green) of the respective karyomorph. Fourth column **(D,H,L,P,T,X)**: merged images of both genomic probes and DAPI staining. The common genomic regions for male and female are depicted in yellow. Bar = 10 μm.

**Figure 4:**
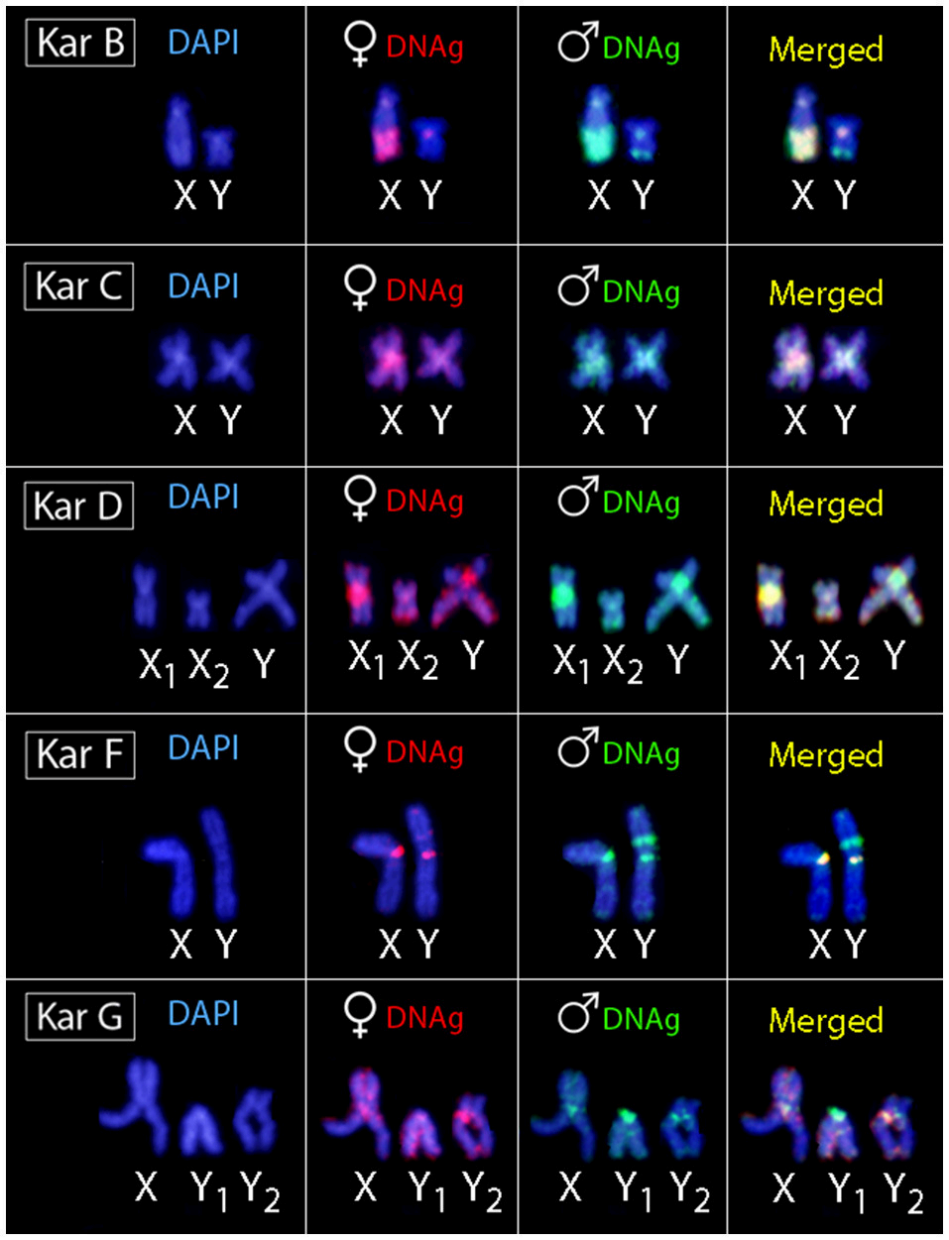
Sex chromosomes of *H*. *malabaricus* karyomorphs B, C, D, F and G after CGH procedures. First column: DAPI images; Second column: hybridization pattern using the female-derived probe (red); Third column: hybridization pattern using the male-derived probe (green); Fourth column: merged images of both genomic probes and DAPI staining. Common genomic regions for male and female are depicted in yellow.

Regarding karyomorph A, no exclusive male-specific regions were identified on male chromosome complement (Figures 3A–D). In male chromosome spreads of karyomorph B, CGH enabled to recognize male-specific region located terminally on the long arms of the Y chromosome (Figures 3E–H). In karyomorph C, unlike the biased accumulation of repetitive DNAs in the X pericentromeric region (Cioffi and Bertollo, 2010), a slight binding preference for the male-derived probe to the pericentromeric region of Y chromosome was evidenced (Figures 3I–L). Female-derived probe produced only a faint hybridization signal in such region, while both probes matched equally the large heterochromatic segment located in the pericentromeric part of the X chromosome. Accordingly, CGH procedure failed to detect any sex-specific region on male chromosomes of karyomorph D (Figures 3M–P). In karyomorph F, a prominent interstitial band on the metacentric Y chromosome was also enriched with male-specific sequences, although a concurrent faint hybridization signal produced by the female-derived probe was also apparent (Figures 3Q–T). CGH on male preparations from karyomorph G unmasked a clear male-specific region covering the short arms of Y_1_ chromosome (Figures 3U–X). The summary of observed intra-karyomorph CGH patterns is given in Figure 4.

## Discussion

### Genomic diversity among karyomorphs

Sex chromosome systems in *H*. *malabaricus* display only male heterogamety and therefore inter-karyomorph genomic comparisons between males were supposed to be informative in indicating their interrelationships. Hence, the male-derived gDNA of reference karyomorph B was probed on chromosomes of karyomorphs A, C, D, F, and G. Even though the B-derived probe showed lower affinity to chromosomes of other karyomorphs, the overall pattern of these experiments was relatively similar. In all experiments, both genome-derived probes showed preferred accumulation to chromosome regions previously identified as C-bands and C_0_t-1 DNA hybridization sites (Born and Bertollo, 2000; Cioffi and Bertollo, 2010; Cioffi et al., 2010, 2011a), documenting their repetitive DNA content. However, despite less intense, hybridization signals were also apparent along the rest of chromosomal material. Our findings are in line with the general patterns observed in previous GISH/CGH-based reports (e.g., Traut and Winking, 2001; Valente et al., 2009; Koubová et al., 2014; Altmanová et al., 2016) in the sense of biased hybridization in heterochromatic regions and point to the fact that even high amount of C_0_t-1 DNA is insufficient to outcompete highly repetitive (heterochromatic) regions. Given that the resolution of the CGH procedure predominantly relies on the presence of species-specific (or sex-specific) repetitive sequences, together with the evolutionary distance of the compared genomes (Kato et al., 2005; Chester et al., 2010), our overall results indicate that karyomorphs of *H*. *malabaricus* are closely related, but with divergences among their genomes, yielding a range of non-overlapping karyomorph-specific signals. Remarkably, the B-derived probe displayed the lowest degree of hybridization correspondence with karyomorphs D and F, suggesting the ongoing processes of sequence divergence. These findings are indicative of ongoing evolutionary processes driving the divergence and possibly also speciation within *H. malabaricus* populations, facilitated by the sedentary lifestyle of these fishes (as discussed in detail further in the text).

An array of molecular and cytogenetic methods, including DNA barcoding and phylogeographic approaches, have already led to the hypothesis that *H. malabaricus* likely represents a “species complex,” with several undescribed species (Bertollo et al., 2000; Santos et al., 2009; Cioffi et al., 2012; Marques et al., 2013). Furthermore, different karyomorphs with a sympatric or syntopic occurrence were found to lack any hybrid forms, as proven by cytogenetic and RAPD (random amplified polymorphic DNA) analyses (Dergam et al., 1998, 2002; Bertollo et al., 2000), yet a sporadic case of hybridization followed by an elevation of the ploidy level was already reported (Utsunomia et al., 2014).

When compared to other members of the Erythrinidae family, similar degree of cytotaxonomic diversity can be found in *Erythrinus erythrinus* and *Hoplerythrinus unitaeniatus* groups (Bertollo, 2007; Cioffi et al., 2012; Rosa et al., 2012; Martinez et al., 2015, 2016), while—in stark contrast—the *Hoplias lacerdae* species complex exhibits highly conserved karyotype structure (Blanco et al., 2011; de Oliveira et al., 2015). It is likely that the chromosomal diversity inside some Erythrinidae species might be associated with their species-specific lifestyles. In this sense, because *H*. *malabaricus, E. erythrinus*, and *H. unitaeniatus* appear to constitute small and restricted populations, with low vagility (Blanco et al., 2011), they experience a higher rate of stochastic fixation of chromosome rearrangements and, consequently, an elevated evolutionary genome dynamism that might contribute to speciation and/or local adaptation processes (see King, 1993; Faria and Navarro, 2010 for an exhaustive discussion). It is therefore remarkable that the three *H*. *malabaricus* karyomorphs with more restricted geographic distribution, i.e., the B, D and G ones, possess morphogically recognizable sex chromosomes (Bertollo et al., 2000; Cioffi et al., 2012).

### Intra-karyomorph genomic diversity and male-specific sequences

Based on data available from the last comprehensive fish karyotype overview (Arai, 2011), so far only 5% of karyologically analyzed actinopterygian fish species possess heteromorphic sex chromosomes. However, this is very likely an underestimation, with many other well-differentiated or even nascent sex chromosome systems still awaiting their discovery, especially when taking into account that homomorphic (i.e., cytologically indistinguishable or hardly detectable) sex chromosomes are thought to be frequent in fishes (Mank and Avise, 2009; Schartl et al., 2016). Within the Erythrinidae family, three different simple or multiple sex chromosome systems in advanced and/or nascent evolutionary stages have been reported among both *E*. *erythrinus* and *H*. *malabaricus* karyomorphs (reviewed in Cioffi et al., 2012), where males possess always the heterogametic sex.

Within *H*. *malabaricus* group, karyomorph A is characterized by 2*n* = 42 for both males and females, without an apparent sex chromosome system, while karyomorph B, though also with 2*n* = 42 for both sexes, exhibits a well-differentiated ♀XX/♂XY sex chromosome system. In this case, the subtelocentric large X chromosome is clearly distinguished from the small-sized metacentric Y, in addition to presence of a conspicuous heterochromatic block distally located on its long arms (Born and Bertollo, 2000; Cioffi et al., 2010). Previous repetitive DNA mapping and WCP data indicate that such sex chromosome system is likely derived from a proto-sex chromosome (the 21st pair of karyomorph A) due enrichment in several types of DNA repeats confined to only one of the homologs, namely the X chromosome in karyomorph B (Cioffi et al., 2010, 2011a,c, 2013). However, CGH procedures did not reveal any sex-specific region in karyomorph A, probably due to low level of sex-specific repetitive DNA divergence or due the small size of the sex-determining region, remaining below the detection limit of the CGH method (that ranges approximately between 2 and 3 Mbp; Schoumans et al., 2004). Theoretically, alternate mechanisms of sex determining region creation such as, e.g., epimutation-coupled recombination suppression (as eloquently discussed in Ezaz and Deakin, 2014) cannot be entirely ruled out and these, again, may have gone undetected through CGH. Finally, the possibility that the sex-determining region in this karyomorph is completely absent seems equally likely. However, the frequent changes of master sex determining genes repeatedly observed in fishes, especially in XX/XY systems (Heule et al., 2014; Martínez et al., 2014), favor the former view as the new non-recombining region is needed to be established again. In support of this view, in several another fish species, the sex determining regions might be very tiny (reviewed in Schartl et al., 2016), with the extreme case of fugu genome, *Takifugu rubripes*, where the Y-specific sex-determining gene differs from the homologous region on the X chromosome by a single non-synonymous substitution (Kamiya et al., 2012). Moreover, even in the platyfish, *Xiphophorus maculatus*, with genetically defined sex chromosomes, no visible differences between X and Y were evidenced after CGH (Traut and Winking, 2001), similarly to what had been occasionally observed in some other animals (Koubová et al., 2014; Altmanová et al., 2016; Green et al., 2016; Gazoni et al., 2018). In yet another case, however, CGH proved to be resolute even in sex chromosomes of a very young age (Montiel et al., 2017). Finally, we cannot entirely exclude the possibility that bright fluorescence from major rDNA loci located on several chromosomes of the karyomorph A complement, most probably including the pair no. 21, could disable the detection of the hypothetical sex-determining region in its vicinity. Maybe a finer-scale approach such as BAC FISH mapping of specific candidate genes identified based on utilization of recent genome sequencing approaches and corresponding bioinformatic tools, togehter with other related “state-of-the-art” technologies may shed more light on this issue (for examples in fishes, see: Reichwald et al., 2015; Portela-Bens et al., 2017; Sutherland et al., 2017; Liu et al., 2018).

A male-specific region confined to a distal part of the long arms of Y chromosome was identified in the karyomorph B corresponding to the location of a constitutive heterochromatin block previously described (Born and Bertollo, 2000). The position of this region is noteworthy since a large NOR is found in the corresponding homologous region on the X chromosome, leading to the considerable size difference between both sex chromosomes (Born and Bertollo, 2000). Therefore, we are dealing here with an unusual situation, resembling the findings in weakly electric fish *Eigenmannia virescens* (de Almeida-Toledo and Foresti, 2001; Henning et al., 2008) or in the snake eel *Ophisurus serpens* (Salvadori et al., 2018), where the accumulation of rDNA and other repetitive DNAs occurs also on the X instead of Y chromosome. In our specific case, the sex-specific region is present on the corresponding C-positive but NOR-negative (Born and Bertollo, 2000; Cioffi et al., 2010) region on the Y chromosome. It is likely that the differential accumulation of repetitive DNA sequences might have decreased the recombination rate between the sex pair due to their delayed pairing during meiosis (Griffin et al., 2002). Alternatively, the co-amplification of the NOR region with other repetitive DNA sequences on the X chromosome can be viewed as a consequence of the whole differentiation process of the sex pair, helping to buffer the absence of functional rDNA copies on the Y chromosome. In fact, it is noteworthy that the NOR on the X chromosome is always genetically active (Born and Bertollo, 2000). The growing number of reports pointing to sex chromosome-specific NORs (see Kawai et al., 2007; Badenhorst et al., 2013; Yano et al., 2017 for references) possibly indicates that such regions might have played a more relevant role in nascent sex chromosome evolution than currently known.

In karyomorph C (2*n* = 40, for both sexes), the nascent and morphologically undifferentiated XY sex chromosomes were formerly evidenced by a small accumulation of repetitive DNAs occurring exclusively on the X chromosome (Cioffi and Bertollo, 2010). Here, it is likely that these newly emerging sex-related elements have not had the necessary evolutionary time to evolve and hence accumulated low proportion of tentatively Y-specific sequences Despite that, we cannot rule out that some differentiation in the hybridization pattern of both genomic probes in the pericentromeric region of the nascent Y chromosome is caused by a copy number variation of interspersed repetitive sequences between the sex chromosomes. Finally, it is worth mentioning that B-derived gDNA probe displayed strong binding to this region (on both X and Y) in the inter-karyomorph experiment (see Figures 2F–H), suggesting certain degree of shared sequences, but the extent of overlap with C-derived probe was not absolute.

Karyomorph D (2*n* = 40 in females/39 in males) is characterized by a X_1_X_2_Y multiple sex chromosomes system, where the neo-Y originated via a tandem fusion between the nascent Y chromosome and one autosomal homolog corresponding to the pair No. 20 of karyomorph C (Bertollo et al., 1997; Cioffi and Bertollo, 2010). Indeed, such origin was also confirmed by additional data from inter-karyomorph chromosome painting and mapping of several repetitive DNA classes (Cioffi et al., 2009, 2011b,c). Previous studies on male meiosis showed stabilized pachytene sex trivalents, as well as asynapsis in the region of presumed sequence divergences (Bertollo and Mestriner, 1998), thus pointing to a putative sex-specific region. In favor of this scenario, Rosa et al. (2009) reported noticeable alterations in location of constitutive heterochromatin and 18S rDNA sites on the neo-Y chromosome, indicating that pericentric inversions probably have also taken place in the early process of the sex-specific chromosome differentiation. However, although in karyomorph C, a slight binding preference for the male-derived probe to the pericentromeric region of Y chromosome was observed, our CGH data did not reveal any conspicuous Y-specific region in the neo-sex chromosome system of karyomorph D. In this sense, while in karyomorph D the recombination arrest and the establishment of the stable multiple sex chromosomes was most likely achieved by chromosomal rearrangements, in karyomorph C the accumulation of repetitive DNA sequences seems to have a central role in triggering the differentiation of the nascent XY sex system (Bertollo et al., 1997; Cioffi and Bertollo, 2010).

Karyomorphs E, F, and G were supposed to be closely related (Bertollo et al., 2000). Although the karyomorph E (2*n* = 42) was not sampled in this study, our results confirmed previous findings in karyomorphs F (Freitas et al., 2017) and G (Oliveira et al., 2018). More specifically, karyomorph F (2*n* = 40, for both sexes) was found to exhibit a nascent XY sex chromosome system, where the male-specific content was highlighted as a prominent interstitial heterochromatic block on the large metacentric Y chromosome, coincident with several microsatellite motifs and retrotransposons (RTEs) (Freitas et al., 2017, present study). Importantly, as the faint hybridization signal produced by the female-specific probe was allocated also within this region, we witness here a bit similar situation to that found in karyomorph C. If we admit that slightly preferred accumulation of male-exclusive sequences in pericentromeric region of the Y chromosome in males of karyomorph C might be related to the early stage of sex-determining region formation, the observed pattern in karyomorph F may reflect a later phase of similar process. At this stage, the accumulation of repetitive DNA in the Y-specific region in karyomorph F probably involves also the portion of sequences that are common for both sexes.

In contrast to karyomorph F, the sex chromosome system found in karyomorph G (2*n* = 40 in females/41 in males) is characterized by presence of XY_1_Y_2_ chromosomes, where the unusual acrocentric Y_1_ element carries the male-specific region, enriched with several different types of repetitive DNAs including 5S rDNA (Oliveira et al., 2018), hence strengthening the view discussed above. As initially proposed by Bertollo et al. (2000) and confirmed by the recent findings (Oliveira et al., 2018), the emergence of sex chromosomes in karyomorph G proceeded through a tandem fusion involving chromosomes from two different pairs that might be tentatively assigned to specific pairs in karyomorph E—a hypothetical ancestral karyotype to both F and G karyomorphs. Importantly, while the tandem fusion was fixed in heterozygous condition in karyomorph G as only one homolog from each pair underwent this rearrangement (hence resulting in unpaired large-sized metacentric X chromosome complemented with the remaining unfused Y_1_ and Y_2_ chromosomes in males), in karyomorph F both homologs from the mentioned pairs gave rise to two large-sized metacentric chromosomes, the X and Y ones (Freitas et al., 2017; Oliveira et al., 2018, this study). Noteworthy, the XY_1_Y_2_ system of the karyomorph G differs from the X_1_X_2_Y neo-sex chromosomes of karyomorph D in the way that a sex-determining region is clearly detectable by CGH only in the former case, indicating a different evolutionary stage between such sex systems. All these findings in karyomorphs F and G, i.e., (i) shared homology between their sex chromosomes, pointing to a common origin through tandem fusion and (ii) lack of homology between multiple sex chromosomes found in karyomorphs D and G, are supported also by recent Zoo-FISH experiments (Oliveira et al., 2018).

In summary, our findings support trends in teleost fishes concerning the independent and repeated evolution of sex chromosomes regardless their phylogenetic relationships (Devlin and Nagahama, 2002; Woram et al., 2003; Schartl, 2004; Mank et al., 2006; Mank and Avise, 2009; Cioffi et al., 2013). It has been shown that the sex chromosome systems and/or the stage of their differentiation may differ evidently not only among closely-related species, but also among different populations of the same species (Takehana et al., 2007; Ross et al., 2009; Zhou et al., 2010; Cioffi et al., 2012; Cnaani, 2013). Such an exceptional sex chromosome variability could be possibly associated with the high plasticity and dynamics of teleost genomes (Ravi and Venkatesh, 2008), a feature usually assigned to a specific whole-genome duplication (TSGD) that occurred at the base of teleostean radiation (Hurley et al., 2007). As a consequence, duplicated redundant copies of different genes might have evolved into master sex-determining genes (Matsuda et al., 2002; Nanda et al., 2002), thus leading to emergence of distinct sex chromosomes in different evolutionary lineages (Schartl, 2004; Mank and Avise, 2009). The outstanding pace of sex chromosome turnover is, however, commonly observable also in other cold-blooded vertebrates such as amphibians and reptiles, hence a number of alternative hypotheses about the evolutionary forces standing behind this phenomenon have already been proposed (for recent reviews and in depth discussion, see Mank and Avise, 2009; Kitano and Peichel, 2012; Kikuchi and Hamaguchi, 2013; Bachtrog et al., 2014; Brykov, 2014; Pokorná et al., 2014; Pennell et al., 2015; Schartl et al., 2016). In a broader context, handful of studies have provided direct evidence that the emergence of sex chromosomes, or even the sex chromosome turnover itself, might play a major role in reproductive isolation promoting evolutionary divergences and eventually speciation (e.g., Kitano et al., 2009; Nguyen et al., 2013), which is evidently the case for *H. malabaricus*.

## Conclusion

Our data provided additional layer of evidence about the status of the taxon *H. malabaricus* and corroborated previous studies in the conclusion that it includes taxonomically distinct species. The CGH procedures proved to be very useful in detecting the hidden biodiversity in this fish group, as they have opened novel views and widen our understanding of the ongoing processes of inter-karyomorph genome differentiation, as well as the amazing variety of sex chromosome systems inside this fish group. Besides, our approach not only uncovered the male-specific regions on the sex chromosomes, but also confirmed different trajectories of the sex chromosome evolution. Future studies using high throughput sequencing will be applied in microdissected sex chromosomes for furthering our understanding of sex determination in this species complex and its possible link with the speciation process.

## Author contributions

AS: Designed the study, performed the experiments, and drafted the manuscript. CY, TH, and EdO: Performed the experiments and drafted the manuscript; PR and LB: Drafted and revised the manuscript; MC: Designed the study drafted and revised the manuscript. All authors read and approved the final version of the manuscript.

### Conflict of interest statement

The authors declare that the research was conducted in the absence of any commercial or financial relationships that could be construed as a potential conflict of interest.

## Abbreviations

2*n*: iploid chromosome number
CGH: comparative genomic hybridization
DAPI: 4′,6-diamidino-2-phenylindole
dUTP: 2′-Deoxyuridine-5′-Triphosphate
FISH: fluorescence *in situ* hybridization
gDNA: total genomic DNA
m: metacentric chromosome
NFDM: non-fat dried milk
NOR: nucleolar organizer region
PCR: polymerase chain reaction
rDNA: ribosomal DNA
SD: sex determination
sm: submetacentric chromosome
TEs: transposable elements
WCP: whole chromosomal painting.
